# Challenges in evaluating risks and policy options around endemic establishment or elimination of novel pathogens

**DOI:** 10.1016/j.epidem.2021.100507

**Published:** 2021-11-17

**Authors:** C. Jessica E. Metcalf, Soa Fy Andriamandimby, Rachel E. Baker, Emma E. Glennon, Katie Hampson, T. Deirdre Hollingsworth, Petra Klepac, Amy Wesolowski

**Affiliations:** a Department of Ecology and Evolutionary Biology, Princeton University, Princeton, NJ, USA; b Princeton School of Public and International Affairs, Princeton University, Princeton, USA; c Institut Pasteur de Madagascar, Antananarivo, Madagascar; d Princeton High Meadows Environmental Institute, Princeton University, Princeton, NJ, USA; e Disease Dynamics Unit, Department of Veterinary Medicine, University of Cambridge, Cambridge CB3 0ES, UK; f Institute of Biodiversity, Animal Health & Comparative Medicine, University of Glasgow, Glasgow, UK; g Big Data Institute, Li Ka Shing Centre for Health Information and Discovery, University of Oxford, UK; h London School of Hygiene and Tropical Medicine, London, UK; i Johns Hopkins Bloomberg School of Public Health, Johns Hopkins University, Baltimore, MD, USA

**Keywords:** Endemic, Epidemic, Mathematical model, Elimination, Emergence

## Abstract

When a novel pathogen emerges there may be opportunities to eliminate transmission - locally or globally - whilst case numbers are low. However, the effort required to push a disease to elimination may come at a vast cost at a time when uncertainty is high. Models currently inform policy discussions on this question, but there are a number of open challenges, particularly given unknown aspects of the pathogen biology, the effectiveness and feasibility of interventions, and the intersecting political, economic, sociological and behavioural complexities for a novel pathogen. In this overview, we detail how models might identify directions for better leveraging or expanding the scope of data available on the pathogen trajectory, for bounding the theoretical context of emergence relative to prospects for elimination, and for framing the larger economic, behavioural and social context that will influence policy decisions and the pathogen’s outcome.

## 1 Introduction

In the extremes, there are two possible fates for a novel pathogen: elimination, or endemicity. The coronavirus that emerged in 2003, SARS-CoV, is an example of global elimination, or ‘eradication’ (Klepac et al. 2013). Stringent international control and containment efforts, aided by clear symptomatic presentation combined with extremely limited asymptomatic transmission allowed the number of human infections with SARS-CoV to be driven down to zero. The last known case was caused by spillover from a palm civet in 2004 (Wang et al. 2005). At the other extreme, currently circulating influenza A viruses derive from the strain that emerged during the 2009 influenza pandemic (Bedford et al. 2015), and are endemic, or present for at least part of the year most years, all around the globe. Such continuous presence, also termed endemicity, has been suggested as a possible fate from the SARS-CoV-2 pandemic (Lavine et al., 2021), noting however, that countries with strong, early public health responses have achieved local elimination.

**Fig. 1:**
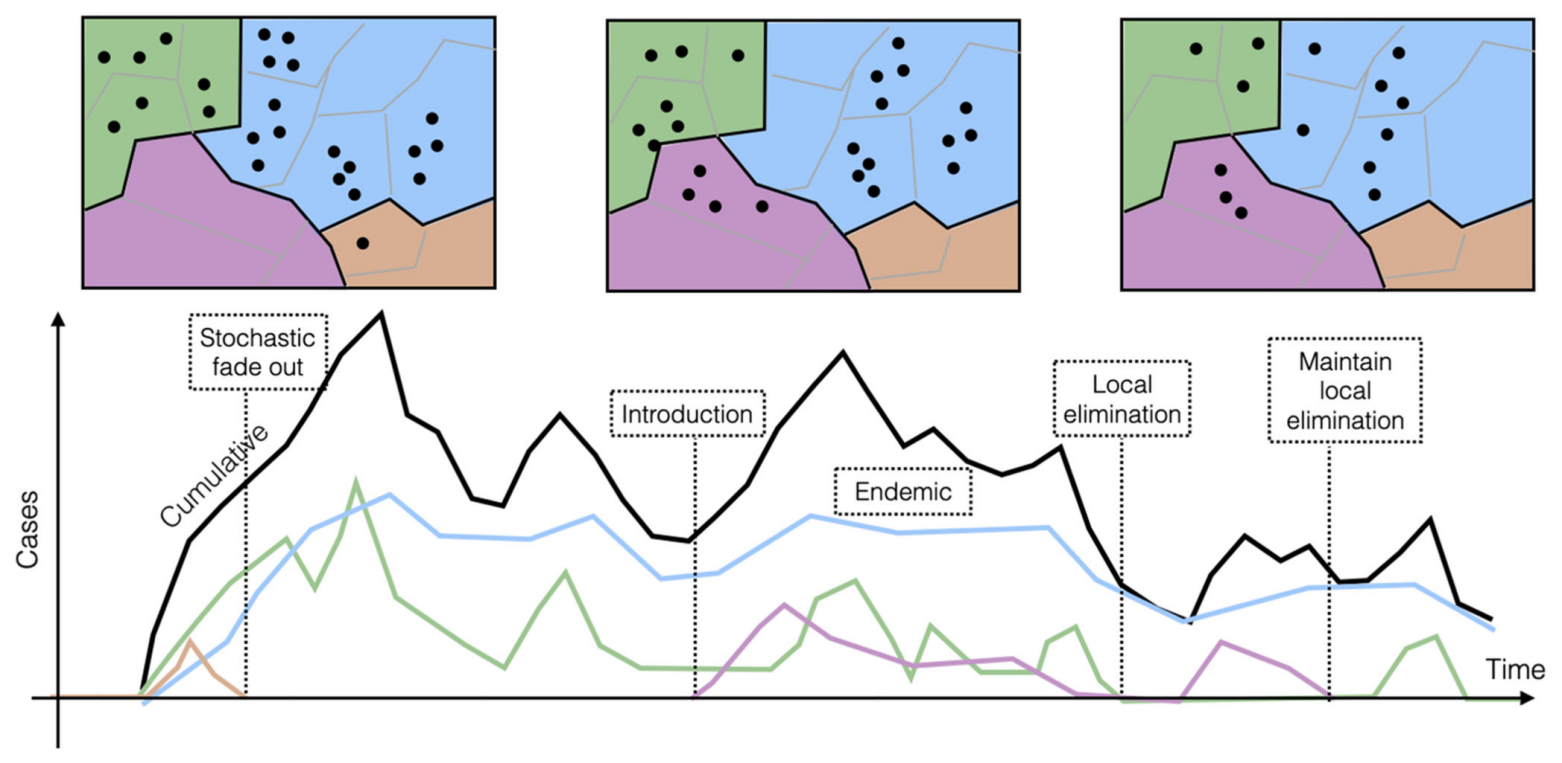
Schematic of endemicity vs. elimination for an emerging pathogen, focusing on a definition of elimination corresponding to absence of transmission, and illustrating the importance of temporal and spatial scale. The top three panels illustrate the spatial pattern of reported cases of an emerging pathogen at three points in time, where filled points indicate the x,y coordinates of each reported case, and color-filled areas indicate different administrative boundaries, such as regions. The bottom panel shows the corresponding numbers of reported cases (y axis) over time (x axis), with the black line showing cumulative cases across all regions, and colored lines showing case totals for each region (y axis), with colors as on the upper panel. In some regions, the pathogen may stochastically fade-out (brown area contains no points after the first panel, and brown line goes to zero on the lower panel) corresponding to elimination (assuming that no infections are missed by case reporting). Alternatively, in some regions, the pathogen might establish continuous circulation (blue and green areas always contain points in the top panels, blue and green lines are always above zero on the lower panel); in others, the pathogen might never arrive, or might rapidly go extinct, but then be reintroduced (purple areas and lines). Thus, the spatial and temporal scales of analysis will define conclusions as to whether the pathogen is endemic or has been eliminated. For example, focusing within the brown area, one might conclude a status of persistent elimination had been achieved. However, if the full spatial extent is considered, pathogen circulation is ongoing at the end of the time-series (black line indicating cumulative cases is above zero at the end of the time-series). (For interpretation of the references to color in this figure legend, the reader is referred to the web version of this article.)

There is room for considerable nuance between these two extremes: local elimination at one spatial scale may vanish under aggregation across spatial scales, while endemicity expands (Fig. 1), and control efforts may result in ‘endemicity’ that corresponds to extremely low incidence levels. Maintaining complete freedom from an infectious agent when it is circulating elsewhere is always challenging (illustrated by many pathogens (Durrheim et al., 2014) including SARS-CoV-2 (Eichler et al., 2021)). Elimination is sometimes used to refer to elimination of disease, or reduction of risk to tolerable levels, rather than elimination of transmission of the pathogen. For example, the World Health Organization (WHO) has a goal to eliminate human rabies mortality by 2030 (Abela-Ridder et al. 2016), rather than a goal of interrupting transmission in the reservoir (even though this is more desirable). Similarly, for some neglected diseases (e.g., leprosy, schistosomiasis, trachoma) WHO has a goal of Elimination as a Public Health Problem (EPHP), corresponding to prevalence and/or incidence falling below a threshold such that morbidity or mortality is considered acceptable at the population level (Toor et al. 2020; Bodimeade et al., 2019). Finally, pathogens whose characteristics have shifted as a result of evolution (e.g., via antigenic drift as for influenza) might or might not be still classified alongside the original emergent strain, leading to different conclusions with regard to endemicity. We focus on the application of these concepts (endemicity, elimination of transmission, elimination as a public health problem) in the context of the emergence of a novel pathogen, i.e., one that has not previously circulated within human populations. We also discuss how the pursuit of these different policy goals will depend both on their feasibility as well as the levels of risk (of re-emergence) and mortality/ morbidity considered tolerable or acceptable, which are highly debatable. Our analysis draws on experience with existing infections and impacts of control efforts, including elimination, or reemergence following lapses in control efforts.

Theoretically, the deterministic requirements for driving an infection to elimination are well established: the reproduction number, R, or the number of new infections per infectious individual, must be pushed to below 1 (Vegvari, 2011). If R_0_ is defined as the number of secondary infections generated from an initial case at the beginning of an epidemic, and thus in an entirely susceptible population, this elimination requirement translates into susceptibles accounting for less than 1/R_0_ of the population, which could be the result of immunization by natural infection, or by vaccination. An alternative useful measure is R_t_, or the reproduction number at time t since the start of the epidemic, which captures the number of secondary infections generated in a population that contains both susceptible and immune individuals, and where control measures such as non-pharmaceutical interventions may have been implemented. R_t_ therefore both changes in value over time and will always be less than R_0_, but if R_t_ can be maintained below 1, again, deterministically, elimination may be achieved (Vegvari, 2011). The absence of an effective vaccine, rapidly waning immunity, or high birth rates eroding immunity in the population, or intense transmission that is hard to diminish, can all make elimination impossible in these deterministic terms (Anderson and May, 1992). Thus, these basic theoretical results provide useful guidance in terms of whether elimination is an achievable policy goal. However, deterministic predictions only provide a partial guide to outcomes in more realistic stochastic and heterogeneous settings - elimination may occur earlier than anticipated by chance; or may be extremely hard to achieve as a result of recurrent reintroductions and metapopulation rescue effects. These important complexities all present open questions in considering the trajectory and appropriate policy responses to novel emergent pathogens (Fig. 1), especially when the range of uncertainties around the characteristics of a novel emergent pathogen are considered.

Establishing the likely trajectory of an emerging pathogen towards the extremes of either endemicity (which may technically include EPHP) or elimination is of fundamental interest, but also has both short and longer term implications for public health. An emerging pathogen that is associated with the risk of a pandemic is perhaps best met by a ‘vertical’ response - i.e., highly targeted, and ideally short-term efforts, across the medical and public health sectors geared entirely towards control of that specific pathogen - which will need to be maintained and potentially intensified if the goal is elimination (Klepac et al. 2013). However, if the pathogen’s trajectory tends towards endemicity, pandemic responses will require ‘horizontal’ integration, i.e., responses must be embedded within the wider health system as part of routine services, rather than as standalone, focused efforts. This will have consequences in terms of resource allocation, and investment in either broad or narrow health system capacity.

Here, we outline challenges for modeling around pathogen emergence in the context of distinguishing between endemicity/elimination in i) contributing to extracting the most information from existing datastreams, or identifying critical areas for expanding data-streams, ii) developing a larger theoretical foundation for characterizing emergent pathogen fate, iii) estimating core epidemiological quantities that provide information about an emergent pathogen’s likely trajectory (including both classic quantities such as R_0_, but also more elusive features such as connectivity), and iv) the larger context of economics, behaviour and policy that impact trajectories towards elimination or endemicity for emergent pathogens.

## 2 Data challenges of future pandemics in the context of endemicity/elimination

The nature of pathogen emergence means, at least initially, considerable unknowns and rapid change, often under crisis conditions. The SARS-CoV-2 pandemic drove many advances in systems for collection of data and improvement of data quality, but gaps clearly remain. Here, we explore how models might contribute to filling these gaps in the context of future pandemics.

### 2.1 Develop generic tools that allow rapid cleaning of data

Around the world, the infrastructure for surveillance proved one of the many aspects of public health that struggled when confronted with the SARS-CoV-2 pandemic. With data-entry reliant on either paper, or unrestricted digital fields, and often little opportunity for training surveillance agents, the opportunity for spelling and other errors proved vast. Rapid deployment of data-cleaning algorithms to resolve, for example the thousands of district names reported in Madagascar into the 114 that actually exist, would have freed up considerable human resources. Development of swiftly deployable probabilistic or fuzzy matching tools (Bradley et al. 2010) across erratic platforms in diverse settings is an important challenge ahead of improvements of surveillance infrastructure.

### 2.2 Develop models that characterize the limits of currently available surveillance data

With clean(er) data in hand, the next set of issues that models can contribute to is in characterizing the limits of surveillance. How appropriate are current data-streams for deriving the distance from elimination (perhaps simply in terms of numbers of cases above zero cases)? Can current data-streams identify whether and where transmission is occurring, with the latter being of particular relevance in establishing whether infection is endemic (e.g., can the original contact of a case be identified), or results from re-introductions (Parag et al., 2021), or novel spillover (Dudas et al. 2018; Kafetzopoulou et al. 2019)? Is undetected transmission likely to be a barrier to elimination (Martinez-Bakker et al., 2015)? Models may be useful in helping to identify or bound the presence of undetected populations where transmission is ongoing (asymptomatics, hard-to-reach populations, etc), and potential reservoir hosts, by integrating across the range of available data (cases, genetic sequences, serology, etc, see Table 1), and identifying contradictions or discrepancies.

### 2.3 Develop novel metrics for elimination

Models might also contribute to extracting the most information possible from available data by development of novel metrics for characterizing distance to elimination. Where cases are hard to track (e.g., for acute infections where the window of opportunity for recording cases is short) an alternative metric for proximity to elimination is the proportion of the population that is susceptible (Metcalf et al. 2020a, 2020b). However, considering either case numbers or proportion susceptible as the target metric neglects the importance of fluctuations over space and time, heterogeneity across populations, and the nuances of the biology of many pathogens.

There is considerable scope for the development of pragmatic metrics that take into account core elements of the biology (e.g., seasonal fluctuations in transmission (Churcher et al. 2014)) to strengthen evaluation of progress towards elimination, leveraging existing data-streams (Table 1). For example, measles case numbers are reported to the WHO annually by every country in the world. As measles vaccination coverage has increased, numbers of cases have fallen, indicating progress towards elimination. However, this progress appears erratic: sudden spikes in cases occur alongside deep troughs. The biology of measles indicates that such ‘post-honeymoon outbreaks’ are expected (McLean and Anderson, 1988). Acknowledging this, the case data can be leveraged to define a canonical pathway towards elimination, and map countries progress towards elimination in a more detailed way - a decline in incidence occurs alongside initially increasing, and then declining variance, further capturing the stochasticity in dynamics as elimination is approached (Graham et al. 2019). Similar combined metrics building on expectations for dynamics built around mathematical models might prove useful across a broader array of pathogen life histories. Given the stochastic nature of epidemiological processes, there is likely to be particular value in leveraging existing theory on critical transitions (Jansen, 2003), which lays down expectations on the frequency distribution of outbreak sizes when R_0_ is below 1; and may be expanded, e.g., to consider the spatial setting (Roy et al. 2014). An added challenge in the context of emerging infections is that data is likely to be sparse and uncertain (many cases may not be counted, case definitions may change (Tsang et al. 2020), etc) and metrics must be designed that are robust to this, alongside realistic framing and careful delineation of the challenges in determining when elimination can be declared (Parag et al. 2020).

### 2.4 Quantify the added value of extended sampling schemes and surveillance strategies

Resources available for surveillance are generally limited. Modeling could be deployed to characterize the added value of, e.g., active sampling in the context of clearly defined surveillance or public health goals, such as locating one case per 100,000 (Chen et al. 2001), targeted genetic sequencing (Holmes et al. 1995), serological surveillance (Mina et al. 2020), etc. Considering the larger question of designing national (or international) surveillance schemes, models could be used to plan the scale of systems adequate not just for the present moment, but for the longer term. The density of sampling will need to keep pace with expected changes in emergence or incidence associated with rapidly changing global conditions, from mobility (Tatem et al. 2012) to climate change (Metcalf et al. 2017), or the amount of contact tracing necessary to maintain elimination (Grantz et al. 2021).

### 2.5 Identify common surveillance needs associated with pathogen characteristics

Effective surveillance for elimination (or to detect cryptic endemicity) will be shaped by the biology of the focal pathogen - for some pathogens, screening for zero cases may not be adequate (Martinez-Bakker et al., 2015), for others interpretation of seronegativity will be complicated by features such as cross-reactivity (Lembo et al. 2013; Rimoin et al. 2010; Lanciotti et al. 2008), for many the impact of contact tracing will be shaped by everything from asymptomatic rates to the distribution of serial intervals (Fraser et al. 2004). Nevertheless, within this diversity, there may be classes of characteristics that emerge as being associated with particularly effective designs for surveillance. Modeling broad pathogen characteristics could illuminate these commonalities. This might, in turn, be valuable in considering how sampling schemes could be optimized across multiple pathogens, potentially of particular relevance as multiplex approaches to sampling (either for pathogens (Finkbeiner et al. 2008) or immune signatures (Mina et al. 2020)) become more tractable.

### 2.6 Identify surveillance needs associated with metapopulation structure and temporal changes

Elimination at one scale may turn to endemicity at another (Fig. 1). Models to delineate the data required to establish whether and what forms of connectivity and metapopulation structure can allow persistence at larger spatial scales despite widespread local elimination is another important and still open question. Genomic sequence data could provide clues to pathogen sources via their relatedness (Worobey et al. 2020), travel/mobility data could establish likely links allowing persistence (Wesolowski et al. 2018), etc (Table 1). Finally, parameters that shape the likelihood of pathogen persistence can often vary, either spatially (e.g., via differences in environmental suitability (Messina et al. 2016)) or temporally (e.g., generation time may be changing in the context of control efforts (Ali et al. 2020)), and these local differences will intersect with the metapopulation context to shape the potential for persistence. Identifying surveillance designs that adequately reflect this variation is another possible and open challenge for modeling endemicity and elimination.

## 3 Challenges in developing the theoretical framework for understanding the likely trajectories of pathogens towards endemicity or elimination

Models have played a central role in establishing the conditions that lead to endemicity or enable elimination (described in the Introduction), but adding realism to this raises a set of challenges.

### 3.1 Develop theory relating to metapopulation context

For many pathogens, at some spatial scale, metapopulation dynamics are likely to play an important role in permitting the transition to endemicity in the face of local extinctions, or facilitating extinction (Fig. 1). Building on core theoretical results (Keeling, 2000; Fox et al. 2017) to reflect synoptic yet realistic features of known systems, such as the structure of connectivity across the hubs of a metapopulation (Mahmud et al. 2021), alongside the pattern of sizes of the connected hubs (from villages to cities, with smaller sizes running a higher risk of extinction by chance (Bjørnstad and Grenfell, 2007)), or the characteristics of travel (Giles et al. 2020) is one important challenge. The importance of these components will be modulated as a function of features of pathogen life history, such as duration of infection (with e.g., little connectivity necessary to guarantee persistence of chronic infections), or potential for recrudescence for apparently recovered individuals (Mbala-Kingebeni et al. 2021), or spill-over from hidden (or known) non-human hosts (Dudas et al. 2018); all of which will reduce the likelihood of local extinction, in ways that could be formally established. Finally, while metapopulation models usually quantify connections between inhabited communities such as villages or cities, increasingly resolved data suggests that heterogeneity within such communities is also likely to be of importance (Dalziel et al., 2013). The difficulties in obtaining sufficiently resolved connectivity data for such settings indicates that another challenge may be in developing phenomenological or semi-mechanistic framings that adequately capture this connectivity.

### 3.2 Develop theory relating to unknown biological features towards endemicity

One important aspect in establishing the trajectory of an emerging pathogen is establishing the probability and characteristics of secondary infection - whether they be rare, associated with little clinical disease, etc. If a vaccine is available, information about the risks and characteristics of infection following vaccination is of similar importance. Since establishing the answers to these questions necessarily takes time (Accorsi et al. 2021) (until sufficient numbers have run the risk of being secondarily infected, little can be said) and is often logistically challenging, one important contribution that theoretical models may provide is a way to explore the potential range of scenarios (Saad-Roy et al. 2020; Lavine et al., 2021) before data is available. More broadly, the longer term consequences of any features of the biology of the pathogen that are hard to pin down during the early phases of the outbreak can be explored using such sensitivity analyses.

A particularly important broad set of unknowns that the SARS-CoV-2 outbreaks has revealed is how the landscape of immunity (or how immune protection is distributed across individuals in the population, where every individual may have either full, partial or no protection) has the prospect to shape immune escape, and, particularly, vaccine escape; alongside selection for increased transmission (Saad-Roy et al. 2021). The development of models that remain tractable, while also formally capturing pathogen phylodynamics within a metapopulation and in the context of shifting selection pressures on immune escape as a function of both vaccination and infection (and potentially even spillback from secondary hosts (Larsen et al. 2021)) is a critical challenge for future work (see also the Chapter on Emergence).

### 3.3 Develop theory relating to feasibility and desirability of elimination

The mortality and morbidity burden of an emergent pathogen, and how these manifest across demographics and environments are likely to determine the degree to which resources are mobilized for elimination. Pathogens with high case fatality rates are likely to be nationally prioritized for elimination in countries that have sufficient resources, because the consequences of endemic circulation will be deemed unacceptable (how this plays out in the global health funding landscape is regrettably less straightforward). Conversely, pathogens that cause only mild disease are less likely to be prioritized, and as a consequence may become endemic. Other pathogen characteristics (e.g., the proportion of transmission that occurs amongst asymptomatic persons, the degree to which transmission can be limited by tractable and acceptable interventions), will shape how tractable and desirable elimination is.

Models can play an important role in characterizing these aspects shaping tractability of local elimination by formally framing the logistics of control (time scale for vaccine development, logistics of roll out, lags in deployment (Townsend et al. 2013) and the underlying biology (duration of immunity, nature of immunity and landscape of selection in the context of immune escape), as well as the extent to which elimination can be maintained (Prada et al. 2017). The latter is important because, even if evidence suggests that the speed required in the response to prevent the establishment of local endemicity is, in fact, tractable, this may not be the most effective public health strategy if elimination is likely to be very easily lost, a question which instantly raises the question of the international context. The development of models that establish tractability and potential for maintenance of local elimination can importantly contribute to discussion around the degree to which elimination (of cases, or infections) is an appropriate goal. But, a vital issue here is in framing models that accurately reflect the inevitably vast range of uncertainties yet contribute to the discussion.

## 4 Challenges in estimating core quantities around endemicity / elimination

Models are clearly crucial in estimating core quantities around pathogen emergence and pandemic response (Metcalf et al., 2020). Many features of estimation relevant to endemicity and elimination are covered elsewhere in this special issue (e.g., on modeling interventions, Kretzschmar et al (2021); on issues around estimation, Swallow et al (2021)). Here, we focus on two features most relevant to endemicity vs. elimination, estimation of parameters relating to emergence and/or elimination, and estimation of parameters during the rapidly shifting phases at the start of an outbreak and in terms of a transition towards endemicity.

### 4.1 Develop approaches to estimate core parameters for elimination and resurgence

In a situation where a novel pathogen has been detected, but its range and potential for spread remain unclear, obtaining rapid yet robust estimates of parameters that will govern rates of local emergence is a critical question (e.g., R_0_, the degree of superspreading, etc.). Minimalist modeling approaches that leverage the most basic of data (e. g., screens for zero cases, or zero infections, deaths (Jombart et al. 2020)) are likely to be important components of an effort in this phase. Extending existing minimalist approaches (for example using hazard-based framing to establish risks of introduction (Bjørnstad and Grenfell, 2007) or branching process analyses to evaluate rates of local spread or probabilities of local elimination (Blumberg et al., 2014) might provide a fruitful direction, alongside extensions that encompass uncertainty in reporting, time-lines likely required for detection of introduction or resurgence (Parag et al., 2021), etc. Relatedly, where theoretical work might establish, for example, patterns of connectivity that make elimination hard to achieve, there will often still be a question of estimating patterns of movement (especially of infected individuals) or recrudescence, or spill-over from reservoir hosts, as these will define the risks of loss of elimination. Efforts to integrate diverse data sources (cases, genetics, mobility, etc) may be a key part of these efforts (Table 1).

### 4.2 Develop approaches to estimate parameters relating to rapidly shifting ground

In the early phases of emergence of a novel pathogen, many things may alter from behaviour, to the public health response, to the distribution of immunity within the population. These changes may be crucial to establishing whether elimination is a possible outcome, but by their nature, estimation may be very complex, since many processes with similar effects will be occurring simultaneously. Identifying ways to leverage existing and diverse data-streams, perhaps across a range of different spatial and temporal frames could be an important future challenge and direction here. For example, reporting rates are very likely to change rapidly during the early phases of emergence of a novel pathogen. Models that integrate epidemiological data with time-varying patterns of testing may provide a way of quantifying this (Subramanian et al., 2021). The converse difficulty of estimating consequences of interventions not yet implemented, especially those with heterogeneous accessibility/uptake across populations is another important challenge, and one that links to the issue of development of models to estimate changing costs of the disease, alongside changing costs of interventions programs along a spectrum from emergent to endemic or elimination (e. g., estimating costs of the ‘last mile’ (Klepac et al. 2013)).

## 5 Challenges in addressing politics, economics, and behaviour around endemicity / elimination

The intersection between politics, economics, behaviours and modeling over the course of the 2020 SARS-CoV-2 outbreak threw up some particularly redoubtable challenges that relate closely to the chapters on economics (Dangerfield et al., 2021) and policy (Hadley et al., 2021) in this Special Issue. An important contribution that modeling might make is to inform decision makers as to the costs and burden of endemicity versus the costs and tractability of achieving elimination, especially in the context of necessary and achievable behaviour change.

### 5.1 Develop modeling approaches that include Epidemiology and Economics

Obtaining accurate estimates of the economic costs of policy decisions and disease impacts is not necessarily straightforward (Metcalf et al., 2020). However, if such costs can be reasonably bounded, models should be able to quantify the outcomes of counterfactual scenarios of elimination versus endemicity (Sandmann et al., 2021). Cost-effectiveness of interventions is typically an important and often challenging component of these framings - for an emerging pandemic the costs of endemic circulation may be outweighed by the detrimental cost of interventions (e.g. school closures (Levinson et al., 2020)) or their disruption to other health services and consequent burden (e.g. other vaccine-preventable diseases (Gaythorpe et al., 2021) or mass drug administration (Hollingsworth et al. 2021)). However, although trade-offs between health and economics were often invoked in the context of policy responses to SARS-CoV-2, robustly characterizing these trade-offs has been elusive, and is likely to be context specific. In some settings, political interests and lobbying colored the discussion, arguably tilting policies in the direction of false economies (Dorn et al., 2021).

The challenge of estimating the costs of both the direct and indirect impacts of the disease and interventions in the shifting context of invasion by a novel pathogen compound the challenge of developing models capable of identifying when investing resources towards achieving elimination is ‘economic’ (Klepac et al., 2011), particularly, as this must include the costs of managing elimination (endpoints may be a moving target, reintroduction is always a risk, etc). There is likely to be particular value in models that discriminate between scenarios where elimination vs. repetitive near-elimination might be most cost effective. Over the longer-term the recurring future benefits of elimination almost always look attractive (Barrett, 2004), but the practical realities of elimination programmes and their projected time horizons can prolong to the point of fatigue. Meanwhile the burden of disease can be minimized through new medicines and tools, potentially making the impacts of infection negligible. Models can plausibly include sensitivity analyses around changes in the mortality rate, alongside the range of considered likely costs and benefits, but the ranges may be hard to bound. Meanwhile, decisions are needed in the near-term, in large part to coordinate global resources and mobilize collective action to enable a controlled trajectory either towards elimination or endemicity, but through choice rather than circumstance. Models have a role to play in laying the landscape to guide these decisions, but, as ever, a critical challenge is managing communication around the range of uncertainties.

Assuming that the challenge of quantifying costs can be addressed, including such costs into dynamical models is also a clear direction of future research with potential to yield insights into applied questions. For example, inclusion of individual-level decision making around costs of both infection and distancing within dynamical models of infectious diseases can alter incidence trajectories and optimal public health strategies associated with vaccination (Jentsch et al., 2021). It is important that, within this effort, the details of the biology are carefully considered - for example, secondary infections and waning in SARS-CoV-2 can starkly alter optimization/cost minimization relative to ignoring these processes depending on time-lines considered. Granularity in the scale of transmission and the role of stochasticity must also be considered, and where important, encompassed. Modeling countries as well-mixed entities is clearly misleading; and, as rare events can have vast consequences, and extremely disparate futures may be equally likely, stochasticity must be carefully framed.

### 5.2 Develop modeling approaches that include Epidemiology and Behaviour

In the early phases of emergence, before availability of a vaccine, successful elimination for a directly transmitted infection like SARS-CoV-2 hinges on alterations in human behaviour. This, in turn, requires policies or recommendations that guide acceptable behaviour change. Acceptability is driven by both individual decision-making but also flows of information. Internal feedback may shape group behaviour (self-reinforcing social norms (Van Bavel et al. 2020), etc), and feedback may also shape the relationship between individual behaviour and incidence (Weitz et al. 2020), potentially with delays (Arthur et al. 2021). Such processes will shape the dynamics of infectious disease incidence, suggesting that developing quantitative and model-grounded and data-informed (Salathé and Khandelwal, 2011) treatments of these flows might considerably enhance our ability to understand and project pandemic-relevant behavioural changes.

An unexpected challenge that emerged during the 2020 pandemic was intense politicization of epidemic outcomes: “Zero Covid” vs. “herd immunity” and “economic sacrifice” narratives, all of which altered the general population behaviours and norms (and thus strategic public health implementation). Feedback loops in behaviour open the prospect of unstable mixed equilibria - for example, if collaboration promotes further collaboration the prospects for elimination are improved; conversely powerful narratives against elimination promote “cheating” behaviour and anti-elimination policies which further discourage and impede elimination strategies. This complex mix of dynamics rooted in the psychological, social and larger political context requires deeper collaboration between modelers and social scientists, as well as around expertize in public health communication (Van Bavel et al. 2020), and this is an important remaining challenge.

### 5.3 Develop modeling approaches that can identify tractable policies nationally

Some of the most important challenges for informing political decisions around targeting elimination occur upstream of model development. It is essential to identify what can be controlled (politically and economically) and what is beyond control (and thus irrelevant for modeling as an intervention strategy); what spatial scales are relevant, and who the key actors are; what will be acceptable targets for interventions (e.g., closure of borders? physical distancing?) and what will not. Introspection as to how prepared countries actually are in response to a public health crisis, and imagination relative to policies that can be successfully implemented, which could be informed by looking to successful countries for example (Patel and Sridhar, 2020) will greatly enhance the utility of models constructed. Models evaluating the potential of flexible policies that can adapt as new information arrives (including evidence on the effect of current policies) might be a useful direction. Alongside this, acceptable levels of uncertainty in informing decisions, policy and practice must be defined (noting that levels of uncertainty may themselves be uncertain); as well as effective tools for communicating both decisions and uncertainty with the public and understanding how this will translate into acceptability.

### 5.4 Addressing transboundary issues and the global context

Whether elimination can be achieved at the country level, regionally or globally, depends upon coordination of interventions across political boundaries. Given vast inequities in resource availability, the willingness of rich countries to support control efforts in poorer countries is likely to be key, and there are many configurations where this will be to mutual advantage, given the ever present risk of pathogen introduction (Klepac et al. 2016). In a globalized world, metapopulation dynamics might be leveraged to rapidly and economically achieve elimination goals (Ruktanonchai et al. 2020). Models have a role to play in persuading policymakers that looking beyond their national boundaries in solidarity is actually in their self interest.

## 6 Discussion

In recent years, there has been considerable debate around the desirability of elimination targets for high burden endemic infections such as malaria (Feachem et al. 2019; WHO Strategic Advisory Group on Malaria Eradication, 2020). The debate emerges from the intersection of uncertainties around logistics, burden, and the complications of perverse incentives (Lockwood et al., 2014). Emerging pathogens manifest many of these challenges, with the added challenge of uncertainties around pathogen biology itself, as well as uncertainties around control options. As knowledge grows in the early phases of pathogen emergence, many core insights from mathematical modeling of pathogen control can be brought to bear (e.g., lower R0, or overlap between symptoms and transmission will facilitate control and potentially elimination (Fraser et al. 2004)) but vast uncertainties inevitably remain. Bounding the theoretical context of emergence relative to the prospects for elimination is likely to require moving beyond simple models, but identifying the most profitable direction for model refinement remains an active area of research.

The most tractable window for elimination is in the early stages of emergence, before a pathogen establishes transmission across large parts of the globe. Indeed countries that acted early with policies aiming for SARS-CoV-2 elimination reaped the benefits (Oliu-Barton et al. 2021). Following the first epidemic waves however, elimination becomes a much greater technical, and perhaps more critically, political, challenge. Many pathogens today circulate endemically in the more impoverished parts of the world, and in many settings, despite the technical feasibility of elimination goals, decision makers with the power to deploy resources to these ends have yet to make commitments (Lembo et al. 2010). Such failures are also starkly illustrated by the pattern of commitments and investments around SARS-CoV-2 control. Although the speed with which a vaccine was developed and deployed was a remarkable product of global collaboration, equitable delivery to mitigate pandemic impacts (not necessarily aiming for elimination) is a much more complex societal challenge. If the opportunity for elimination is not taken early, steering the subsequent trajectory away from endemicity becomes increasingly hard, even if it remains the desired outcome. Whether modeling can be sufficiently fast, accurate and persuasive/believable (at least to policymakers and political leaders) to guide appropriate action in the event of future emerging pathogens is an open question (Sridhar and Majumder, 2020).

## Figures and Tables

**Table 1:** Examples of data-sources, their uses and integration into models, and associated core challenges. That many of the listed data-sources are open-access has been critical to their utility in responding to infectious disease outbreaks.

Type of data	Uses	Integration into models	Challenges
Routine surveillance for cases - laboratory confirmed - suspected - syndromic Examples sources: Healthmap, flutrackers, DHIS2, sentinel systems for ILI and SAR	Estimate parameters (Rt, generation time); effectiveness of interventions; evidence of circulation	Fit both biological parameters and estimates of the impact of interventions (e. g., trajectory matching); verification of elimination	Collation, harmonization, Sensitivity and specificity (especially for syndromic surveillance)
Genetic sequence data *Example sources:* Genbank,GISAID, Nextstrain, Microreact	Infer transmission pathways, pathogen relatedness, distinguish cryptic transmission versus incursions; inferring dynamical/immunological differences between variants	Timing and number of introductions; using variant frequencies/distribution to infer pathogen characteristics/fitness	Speed of pathogen evolution (limits inference of who infected whom e. g. in nosocomial transmission (Abbas et al., 2021)); uneven sampling across populations
Serology *Example sources:* serotracker.com (noting all SARS-CoV-2)	Estimate attack rate/force of infection; susceptibility	Landscape of immunity, i.e. retrospective or prospective pathogen spread	Difficult to collect, variance among assays, waning at initially unknown rates (Takahashi et al., 2021), uncertain (and often hard to resolve) relationship between serology and protection;
Animal reservoir sequencing (or serology) *Possible sources:* Genbank, GISAID, etc	Spillover (and spillback) risk;	Model frequency of spillover/introductions	Hard to sample a wide area
Census based population density, structure by age, etc *Possible sources:* worldpop.org, GPW/SEDAC	Case fatality, morbidity in different settings	Burden, costeffectiveness, spread	Unavailable in some resource poor settings
Timing, location and scope of interventions *Possible sources:* Blavatnik School of Government COVID-19 Government Response Tracker	Rt, and impact of interventions	Cost-effectiveness	Disentangling specific effects of interventions when deployed in combination in different populations/intensities
Remote sensing/satellite imagery *Possible sources: NASA (e.g*. https://neo.sci.gsfc.nasa.gov/), *ESA* (https://earth.esa.int/eogateway/)	Populations at risk, suitable habitat, seasonality of transmission and global range		Climate role may be limited for emerging pathogens
Mobile phone data, social media data *Possible sources:* google mobility (https://www.google.com/covid19/mobility/);	Mobility	Modulation of Rt, responses to policy information	Not necessarily clear that it captures transmission relevant movement; may not be available for critical populations
Social media related information providing a window onto sentiment dynamics *Possible sources:* twitter, facebook (https://dataforgood.fb.com/)	Evolution of social norms, spread of misinformation	Behaviour feedbacks on transmission	Mapping from social media to behaviour not always straightforward
